# 

**Published:** 1842-04

**Authors:** 


### WESTERN JOURNAL

OF

### MEDICINE AND SURGERY.

### EDITORS.

---

DANIEL DRAKE, M. D.,

PROFESSOR OF PATHOLOGICAL ANATOMY AND CLINICAL MEDICINE IN THE MEDICAL INSTITUTE OF LOUISVILLE:

LUNSFORD P. YANDELL, M. D.,

PROFESSOR OF CHEMISTRY AND PHARMACY IN THE SAME:

THOMAS W. COLESCOTT, M. D.

### RECEIPTS

*For the Medical Journal for March*, 1842.

---

| | |
|---|---:|
| Dr. C. J. Blackburne, Frankfort, Ky., | $5 00 |
| S. Willis, Shelbyville, Ky., | 5 00 |
| W. M. M'Neil. Holly Springs, Miss., | 5 00 |
| J. Van Eaton, Mt. Hope, Ala,, | 5 00 |
| J. McRoberts, Campbellsburgh, Ky., | 5 00 |
| T. B. Hindley, Waidsboro, Ky., | 5 00 |
| Drs. Kalston & Stahl, Quincy, Ill., | 5 00 |
| Dr. W. H. Anderson, Bellview, Ky., | 5 00 |
| E. Rees, Henderson, Ky., | 5 00 |
| D. S. Smith, Juliet, Ill., | 5 00 |
| A. H. Slaughter, New Haven, Ky., | 5 00 |
| W. H. Lee, Mexico, Mo., | 5 00 |
| R. B. Harper, Portersville, Tenn., | 5 00 |
| P. Herod, Pleasant Shade, Tenn., | 5 00 |
| Hazlette, Dresden, O., | 5 00 |
| J. W. Fennell, Cottonville, Ala., | 5 00 |
| W. L. Sutton Georgetown, Ky., | 5 00 |
| H. Burritt, Gilead, O., | 5 00 |
| Morrison, Arcadia, Ill., | 5 00 |
| A. O. Ayer, Owensboro, Ky., | 10 00 |
| J. Dunson, Bedford, Ia , | 10 00 |
| J. R. Hamilton, Raleigh, Tenn., | 5 00 |
| J. C. Whitlock, Bellview, Ky., | 5 00 |

